# Multiple CheY Proteins Control Surface-Associated Lifestyles of *Azospirillum brasilense*

**DOI:** 10.3389/fmicb.2021.664826

**Published:** 2021-04-22

**Authors:** Elena E. Ganusova, Lam T. Vo, Tanmoy Mukherjee, Gladys Alexandre

**Affiliations:** Department of Biochemistry and Cellular and Molecular Biology, University of Tennessee, Knoxville, TN, United States

**Keywords:** *Azospirillum*, CheY, chemotaxis, flagella, swarming, surface attachment

## Abstract

Bacterial chemotaxis is the directed movement of motile bacteria in gradients of chemoeffectors. This behavior is mediated by dedicated signal transduction pathways that couple environment sensing with changes in the direction of rotation of flagellar motors to ultimately affect the motility pattern. *Azospirillum brasilense* uses two distinct chemotaxis pathways, named Che1 and Che4, and four different response regulators (CheY1, CheY4, CheY6, and CheY7) to control the swimming pattern during chemotaxis. Each of the CheY homologs was shown to differentially affect the rotational bias of the polar flagellum and chemotaxis. The role, if any, of these CheY homologs in swarming, which depends on a distinct lateral flagella system or in attachment is not known. Here, we characterize CheY homologs’ roles in swimming, swarming, and attachment to abiotic and biotic (wheat roots) surfaces and biofilm formation. We show that while strains lacking CheY1 and CheY6 are still able to navigate air gradients, strains lacking CheY4 and CheY7 are chemotaxis null. Expansion of swarming colonies in the presence of gradients requires chemotaxis. The induction of swarming depends on CheY4 and CheY7, but the cells’ organization as dense clusters in productive swarms appear to depend on functional CheYs but not chemotaxis *per se*. Similarly, functional CheY homologs but not chemotaxis, contribute to attachment to both abiotic and root surfaces as well as to biofilm formation, although these effects are likely dependent on additional cell surface properties such as adhesiveness. Collectively, our data highlight distinct roles for multiple CheY homologs and for chemotaxis on swarming and attachment to surfaces.

## Introduction

Navigating chemical gradients requires motile bacteria to sense and bias their direction of movement using chemotaxis. Motile and flagellated bacteria utilize conserved and dedicated chemotaxis signal transduction pathways to modulate swimming bias in chemical gradients. In *Escherichia coli*, the chemotaxis system comprises a single set of membrane-bound chemoreceptors, chemotaxis histidine kinase (CheA), flagellar-motor binding response regulator (CheY), and scaffolding protein (CheW). Adaptation proteins methylesterase CheB and methyltransferase CheR re-set signaling upon excitation by reversibly modifying membrane-bound chemoreceptors (Levit and Stock, 2002). The majority of motile, flagellated bacterial sequenced genomes indicates the presence of multiple chemotaxis as well as chemosensory (chemotaxis-like) pathways, with the latter displaying non-motility phenotypes such as extracellular matrix formation (Edwards et al., 2011), cyst formation (Berleman and Bauer, 2005; Wu et al., 2011), biofilm formation (Huang et al., 2019), and quorum sensing (Laganenka et al., 2016). In contrast to *E.coli* which possesses a single chemotaxis response regulator CheY to alter the direction of rotation of flagellar motors, the genome of many bacteria encodes for multiple CheY homologs: *Rhodobacter sphaeroides* (Ferré et al., 2004; Porter et al., 2006), *Sinorhizobium meliloti* (Schmitt, 2002), *Rhizobium leguminosarum* (Miller et al., 2007), *Azospirillum brasilense* (Mukherjee et al., 2016, 2019), *Borrelia burgdorferi* (Pitzer et al., 2011), *Vibrio cholerae* (Hyakutake et al., 2005), etc. In some cases, the multiple CheY homologs are encoded within a single chemotaxis pathway (e.g., *S. meliloti*). These CheYs may also be encoded elsewhere on the genome with no apparent genetic link to a particular chemotaxis system (e.g., *B. burgdorferi*; Schmitt, 2002; Pitzer et al., 2011). Why motile bacteria have multiple chemotaxis-related response regulators is not clear. In *R. sphaeroides*, all six CheY homologs are able to bind to the FliM component of the flagellar motor upon phosphorylation, but only one of them is responsible for the flagella motor stopping (Ferré et al., 2004).

The alphaproteobacterium *A. brasilense* are soil motile diazotrophic bacteria able to colonize the roots of diverse plants and promote their growth through phytohormones production and nitrogen fixation (Steenhoudt and Vanderleyden, 2000). *A. brasilense* motility and chemotaxis are important for plant root colonization (Zhulin and Armitage, 1992; Greer-Phillips et al., 2004; O’Neal et al., 2019, 2020). *A. brasilense* cells are motile using a single polar flagellum that allows the cells to swim in liquid media and when the viscosity of the media increases, cells produce multiple lateral flagella, structurally distinct from the polar flagellum, that permit translocation across surfaces by swarming (Moens et al., 1996). The polar flagellum of *A. brasilense* cells rotates in both clockwise and counterclockwise directions, and chemotaxis signaling controls the rotational bias of the polar flagellum in this species (Zhulin and Armitage, 1993; Mukherjee et al., 2019). The *A. brasilense* polar flagellum is comprised of flagellin that is glycosylated (Moens et al., 1995b). The glycosylation on the *A. brasilense* polar flagellin consists of a branched tetrasaccharide with repeated rhamnose, fucose, galactose, and *N*-acetylglucosamine that resemble the LPS *O*-antigen, suggesting that both structures are related (Belyakov et al., 2012). The polar flagellum was suggested to mediate adsorption of *A. brasilense* cells to the roots of wheat plants in a two-step attachment process (Croes et al., 1993): a reversible step that is thought to be mediated by the polar flagellum and an irreversible attachment step that likely involves extracellular polymeric substances. The *A. brasilense* polar flagellum also contributes to biofilm formation *in vitro* and to the stabilization of the biofilm matrix (Viruega-Góngora et al., 2020). The lateral flagella required for swarming are distinct appendages made of proteins unrelated to the polar flagellum, including distinct lateral flagellin, termed Laf1 (Moens et al., 1995a). Lateral flagella are produced when the rotation of the polar flagellum is hindered (Moens et al., 1996), and recent evidence indicates that an extracytoplasmic function (ECF) sigma-factor ultimately regulates lateral flagellar biosynthesis in *A. brasilense* (Dubey et al., 2020).

**Figure 1:**
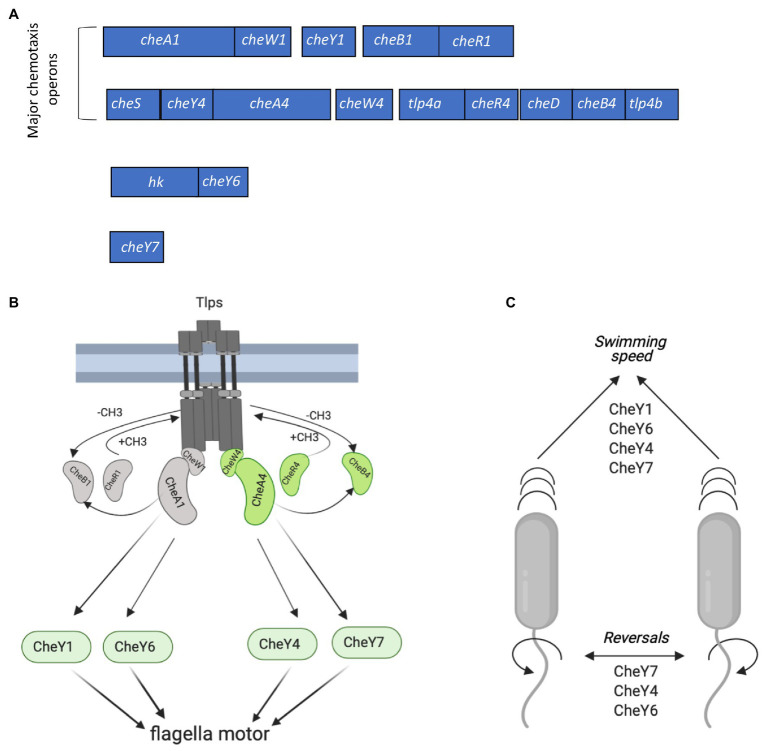
Chemotaxis signaling in *Azospirillum brasilense* and gene clusters encoding CheY response regulators. **(A)** Open reading frames (ORFs) are drawn to scale. The chemotaxis-related genes within each cluster were either previously characterized or identified by homology searches. hk, histidine kinase. Response regulators CheY1 and CheY4 are encoded with each of the two major chemotaxis operons (*che1* and *che4*). CheY6 and CheY7 are encoded elsewhere on the genome. **(B)** Membrane bound chemotaxis receptors (Tlps) are organized in signaling arrays with mixed base plates consisting of CheW1/CheA1 and CheW4/CheA4 proteins. Environmental signals (repellent or chemoattractant) received by Tlps modulate changes in their conformation and autophosphorylation activity of CheA1 and/or CheA4, which ultimately affect the phosphorylation states of flagellar-motor switching response regulators (CheYs). The activity of Tlps is switched off by the addition of the methyl groups chemoreceptor-specific methyltransferase (CheR1 and CheR4) and switched on by the removal of the methyl groups by the chemoreceptor-specific methylesterase, CheB1 and CheB4. Activity of CheB1 and CheB4 depends on autophosphorylation of CheA1 and CheA4. **(C)** Scheme depicting the role of CheYs in the modification of the swimming speed and reversals. Figures in panels **(B,C)** were created using Biorender.com.

Chemotaxis in *A. brasilense* controls the polar flagellum and thus swimming through signaling *via* two different chemotaxis systems, named Che1 and Che4, as well as additional CheY response regulators (CheY6 and CheY7) encoded outside of *che1* and *che4* (Bible et al., 2012; Mukherjee et al., 2016, 2019; Figure 1A). Histidine kinase CheA1 and the response regulator CheY1, both encoded within the *che1* cluster, regulate transient changes in swimming speed during chemotaxis. CheA4 histidine kinase and the CheY4 response regulator, both encoded within the *che4* operon, control the probability of changes in the swimming direction (herein reversals) during chemotaxis (Figure 1B). A *ΔcheY6* mutant has a swimming reversal phenotype similar to that of a *ΔcheY4* mutant, while a strain lacking CheY7 does not display any swimming reversals. All CheY mutants also swim slower than the wild type in the absence of a gradient (Figure 1C; Mukherjee et al., 2019). Genetic evidence and behavioral assays indicate that CheY6 activity is controlled by Che1/CheA1 signaling, and CheY7 activity, a mutant of which phenocopies a *ΔcheA4* mutant, is controlled by Che4/CheA4 signaling (Mukherjee et al., 2019; Figure 1A). Mutants lacking *cheA4*, *cheY4,* or *cheY7* are unable of chemotaxis in spatial gradients of chemoeffectors, while mutants lacking *cheY1* or *cheY6* still display chemotaxis under these conditions (Bible et al., 2008; Mukherjee et al., 2016, 2019). Another feature of the polar flagellum motor of *A. brasilense* is that it undergoes brief swimming pauses, which are distinct from speed increases or reversals. Swimming pause frequency is reduced in strains lacking CheY1, CheY6, and CheY7 but is increased in a strain lacking CheY4, although the mechanism for these differential effects is not known (Figure 1B; Mukherjee et al., 2019). The effects of these CheY homologs on the swimming motility pattern of *A. brasilense* are thought to optimize navigation in the spatially and physically heterogeneous environment of the soil, although this remains to be experimentally demonstrated.

Rotation (or lack thereof) of flagella controlling swimming motility has been implicated in the swim-to-sessile transitions in diverse bacteria, perhaps through the flagellum acting as a “mechanosensor” (Gordon and Wang, 2019; Chawla et al., 2020). Such swim-to-sessile transitions occur during swarming on surfaces, the formation of biofilms, and surface attachment (Guttenplan and Kearns, 2013). Chemotaxis and chemotaxis mutants with different motility biases have been implicated in bacterial social behaviors, promoting cell-to-cell interactions or interaction with eukaryotic hosts (Alexandre, 2015). However, the exact role of the rotational bias of flagella or multiple chemotaxis CheY homologs in these behaviors has been seldom, if at all, addressed. Here, we take advantage of the different effects of *A. brasilense* CheYs (CheY1, CheY4, CheY6, and CheY7) on the rotational bias of the polar flagellum and chemotaxis to examine contributions to behaviors related to swim-to-stick transitions such as swarming, surface (abiotic and wheat) attachment, and biofilm formation. We show that only some of these CheYs (CheY4 and CheY7) but not chemotaxis *per se* induce swarming, and CheYs mediate distinct abiotic surfaces and wheat roots attachment as well as biofilm formation. Together, the findings indicate that CheY homologs contribute to distinct swim-to-stick behaviors.

## Materials and Methods

### Bacterial Strains and Culture Conditions

The bacterial strains used in this study are listed in Table 1. *A. brasilense* strains were cultured in the minimal medium (MMAB; Hauwaerts et al., 2002) or TY (tryptone 10 g/L, yeast extract 5 g/L; Bible et al., 2012); and washed in a chemotaxis buffer [10 mM phosphate buffer (pH 7.0), 1 mM EDTA] as described previously (Stephens et al., 2006). Conjugation was performed on D-plates (8 g/L Bacto Nutrient broth, 0.25 g/L MgSO_4_ 7H_2_O, 1.0 g/L KCl, 0.01 g/L MnCl_2_, 2% agar) and, after conjugation, MMAB with appropriate antibiotics was used for selection of *A. brasilense* transconjugants. The *A. brasilense* wild type (Sp7), mutant strains, and complemented derivatives were grown at 28°C, with shaking. Unless otherwise stated, the antibiotics were used at the following concentrations: 200 μg/ml ampicillin, 30 μg/ml kanamycin (Km), 20 μg/ml gentamicin (Gm), 34 μg/ml chloramphenicol (Cm), and tetracycline (Tc) 5 μg/ml. CheY7-YFP complementation construct was obtained using Gateway cloning (Invitrogen) and the pRH005 vector according to the published protocols (Hallez et al., 2007). *cheY7* gene was amplified using Gateway primers (Table 1) and Sp7 *A. brasilense* genomic DNA as a template. Five microliters of PCR product were run on a 0.8% gel for verification of the insert, and PCR cleanup (Nucleospin Gel and PCR cleanup, Macherey-Nagel™) was performed on the remainder of the PCR product. The resulting PCR product was used for a BP Clonase (Invitrogen™) reaction with the pDONR2.1 vector (Invitrogen™). This reaction was then transformed into *E. coli* Top10 chemically competent cells and plated on Luria broth (LB, 10 g/l tryptone, 5 g/l yeast extract, 10 g/l NaCl) with 50 mg/ml kanamycin. Colonies from these plates were grown in 5 ml of LB with kanamycin (50 μg/ml) and subjected to plasmid purification (Qiagen™). The resulting plasmids were used for the LR Gateway reaction (in the Gateway cloning LR Reaction stands for a recombination reaction between attL and attR sites; Invitrogen™) with the pRH005 plasmid.

**Table 1:** The list of strains and plasmid used in this study.

Strain or plasmid	Description	Reference or source
*A. brasilense* Sp7 *ΔcheY1* *ΔcheY4* *ΔcheY7* *ΔcheY6* *rpoN::Km^r^*	Wild type strain *ΔcheY1*::Km (Km^r^) *ΔcheY4*::Cm (Cm^r^) *ΔcheY7*::Gm (Gm^r^) *ΔcheY6,* markerless *rpoN::Km^r^* in Sp7 (Km^r^)	ATCC 29145 Bible et al., 2008 Mukherjee et al., 2016 Mukherjee et al., 2019 Mukherjee et al., 2019 Milcamps et al., 1996
*E. coli* TOP10	General cloning F-*mcr*A Δ(*mrr-hsd*RMS-*mcr*BC) φ80*lac*ZΔM15 ΔlacX74 *rec*A1 *ara*D139 Δ(*araleu*)7697 *gal*U *gal*K λ^–^ *rps*L (Str^R^) *end*A1 *nup*G	Invitrogen™ Simon et al., 1983
pRK2013 pHRGFP pRH005 pRHCheY4 pRHCheY7 pRK415 pRKCheY1 pRKCheY4 pRKCheY6 pRKCheY7	Helper plasmid for triparental mating (ColE1 replicon, Tra, Km^r^) pBBR1 origin plasmid expressing GFP (Tc^r^) Gateway-based destination vector expressing proteins fused with YFP at the C-terminus, Km^r^, Cm^r^ pRH005 plasmid with CheY4 ORF fused with YFP at the C-terminus (CheY4-YFP) pRH005 plasmid with CheY7 ORF fused with YFP at the C-terminus (CheY7-YFP) Broad host range vector (Tc^r^) pRK415 containing *cheY1* (Tc^r^) pRK415 containing *cheY4* (Tc^r^) pRK415 containing *cheY6* (Tc^r^) pRK415 containing *cheY7* (Tc^r^)	Figurski and Helinski, 1979 Ramos et al., 2002 Hallez et al., 2007 O’Neal et al., 2019 this study Keen et al., 1988 Bible et al., 2008 Mukherjee et al., 2016 Mukherjee et al., 2019 Mukherjee et al., 2019

### Chemotaxis, Swimming, and Swarming Behavioral Assays

For the aerotaxis spatial gradient assay, free-swimming cells from exponentially grown cultures were washed twice with chemotaxis buffer and placed in a 1 mm flat capillary tube (inner dimensions, 0.1 by 2 by 50 mm; VitroCom, Mountain Lakes, NJ, United States). The formation of a band of motile bacteria near the air-liquid interface was observed at 60 s post-introduction into the capillary tube, and the distance between the meniscus and the band was measured. Aerotaxis band formation was recorded using a Nikon microscope with a Nikon Coolpix mounted camera.

For the swimming and swarming assays in Petri plates, a single colony from each strain was inoculated in 5 ml of MMAB medium and grown until OD_600_ = 0.8. The culture was then washed once with modified chemotaxis buffer [10 mM phosphate buffer (pH 7.0)], and 5 μl of the culture was placed on top of 13 g/l Nutrient broth (Fisher Scientific™) solidified with 0.2, 0.3, 0.4, 0.5 0.6, or 0.7% (w/vol) of noble agar (Fisher Scientific™). A swarming time-course assay was conducted using 0.6% noble agar added to MMAB and supplemented with 0.5% Tween-20 since preliminary data indicated its addition promoted reproducible and robust swarming (Wu et al., 2020). The plates were incubated at 28°C for 24–96 h, and the diameter of the expansion rings was measured.

To observe the development of swarming colonies under the microscope, we used the pHRGFP plasmid with constitutive green fluorescent protein (GFP) expression in *A. brasilense* (Ramos et al., 2002). The pHRGFP plasmid was introduced into the wild type Sp7 strain and its *cheY* mutant derivatives by conjugation. Three hundred microliters of the swarming medium [MMAB with 0.6% (w/v) noble agar and supplemented with 0.5% Tween-20] were placed in the well of an EISCO Concavity Microscope slide (Fischer Scientific™). Two microliters of the cell culture, prepared as for the swarm plates assay above, were diluted to an OD_600_ = 0.8 and placed at the center of the swarming medium. Under these conditions, swarming of the cells was observed using a GFP filter, 4x objective mounted to a Nikon ECLIPSE 80i fluorescence microscope with a Nikon CoolSnap HQ2-cooled charge-coupled device (CCD) camera and photographed in 2, 6, and 24 h. The experiment was conducted in triplicate. Whole colony swarming fluorescence images were obtained using a Leica MZ167A dissecting scope equipped with a GFP fluorescence filter. Leica application suite software was used for the image collection. To observe the formation of cell clusters in swarming colonies of *A. brasilense* Sp7 and a *rpoN::Km^r^* harboring a pHRGFP plasmid, slides were prepared the same way as described above except a cover slip was placed on the top of the agar inoculated with cells. Clusters were observed using a GFP filter, 100x objective mounted to a Nikon ECLIPSE 80i fluorescence microscope with a Nikon CoolSnap HQ2-cooled charge-coupled device (CCD) camera and photographed at 2, 6, and 24 h post inoculation.

### Flagella Staining

Flagella staining was performed on cells grown in liquid medium (swimming) or collected from swimming/swarming MMAB media made with 0.2–0.7% (w/vol) agar plates after 48 h incubation and stained using Alexa Fluor™ 488 NHS Ester (Succinimidyl Ester; Fisher Scientific™) as described in (Gunsolus et al., 2014) with slight modifications. Briefly, cells were resuspended in 75 μl of in phosphate buffer saline (PBS; 137 mM NaCl, 2.7 mM KCl, 10 mM Na_2_HPO_4_, 1.8 mM KH_2_PO_4_) supplemented with 1 mM EDTA and 0.5% Brij^Tm^-35 detergent (Fisher Scientific™) to avoid cell-to-cell adhesion at OD_600_ = 1 and 25 μl of 1 M sodium bicarbonate bicarbonate buffer was added to the cell suspension to stabilize the pH. A 1 μl of 0.5 mg ml^−1^ Alexa Fluor 488 carboxylic acid succinimidyl ester (Fisher Scientific™) in DMSO (Sigma Aldrich) was added. The resulting suspension was incubated in the dark for 1 h at room temperature with frequent mixing. The suspension was then centrifuged at 4,000 rpm for 3 min, the supernatant was discarded, and the cell pellet was washed with 500 μl of PBS. Cells were mounted on an agar pad (1% low melting point agarose in PBS) and covered with a glass coverslip, and left on the bench for 10 min. Images were taken using a 63x objective with oil immersion mounted to a Leica SP8 with White Light Laser Confocal System; Leica, Wetzlar, Germany). Images were collected using a 488-nm excitation Argon ion laser with an emission maximum at 517 nm.

### Measurements of Cell Size

Cells were washed once with PBS buffer and resuspended in TBAC buffer [PBS containing 1 mM EDTA and 0.01% (v/v) Tween 20] to avoid the formation of bacterial aggregates (Alves et al., 2017). Cell sizes were measured using the Prism 8 program for a minimum of 60–100 cells per sample taken from at least four different fields of view. Confocal microscopy (Leica SP8 White Light Laser Confocal System) images were taken at random fields of view. Several images were collected for each experiment. For cell length measurements, all cells within the field of view were measured from one cell pole to the other at the longest axis.

### Western Blotting, Coomassie Staining, and Flagella Glycosylation Staining

For isolation of polar flagella, each strain was grown to the mid-log phase (OD_600_ = 0.7–0.8) in liquid MMAB. Cells were pelleted for 3 min at 4,600 rpm using a tabletop Eppendorf centrifuge and washed once with 1 ml of PBS buffer. The resulting pellet was resuspended in 150 μl of 1x Laemmli buffer (4% SDS, 10% beta-mercaptoethanol, 20% glycerol, 0.1 M Tris pH 6.8, and 0.005% of bromophenol blue) in PBS. Cells were vigorously vortexed for 1 min and spun down for 15 min at 4°C and 13,000 rpm using a tabletop Eppendorf centrifuge. The supernatant was collected and heated for 5 min at 65°C to denature proteins. For isolation of lateral flagella, each strain was grown on the top of the swarming medium (MMAB supplemented with 0.6% of noble agar and 0.5% of Tween-20) for 48 h at 28°C. Cells were then scraped from the plate and resuspended in PBS. Flagella isolation was done as described for the isolation of polar flagella. Twenty microliters of isolated flagellins were loaded on SDS-PAGE gels (8% resolving gel for polar flagellin analysis and 12% resolving gel for lateral flagellin detection). Mini-Protean gel system was used for protein separation (Bio-Rad™). The gel ran at 120 V for 90 min. The gel was then transferred to a 0.45 μm hydrophobic polyvinylidene difluoride (PVDF) transfer membrane (Immobilon) using a wet transfer apparatus (Bio-Rad). The transfer ran at 90 V for 1 h and 10 min. The membrane was blotted for 40 min in 5% milk in Tris-buffered saline (TBS; 6.05 g/L Tris, 8.76 g/L NaCl, pH 7.5) supplemented with Tween-20 (0.1%; TBST). After blocking, the membrane was incubated with primary polyclonal anti-polar and anti-lateral flagellin antisera (Alexandre et al., 1999) in TBST at 1:1,000 for 16 h at 4°C with agitation. The membrane was washed twice with 5% milk in TBST, twice in TBST, and twice in TBS. The membrane was then incubated with secondary anti-rabbit antibodies, diluted to 1:10,000 in TBS for 1 h, and washed again with the solutions mentioned above. Lateral protein production was quantitated using Fiji ImageJ (NIH). Coomassie blue dye (2 g/L of water) was used to monitor total proteins loaded. SDS-PAGE gels were de-stained using a mix of H_2_O, methanol, and acetic acid detected using the Glycoprotein Staining Kit (Thermo Scientific™ Pierce™) according to the manufacturer’s manual. Briefly, *A. brasilense* cells were grown in flasks with 25 ml of MMAB medium with shaking at 175 rpm, at 28°C. Cell cultures were spun down at 3,000 rpm in a 50-ml Falcon tube, cell pellets were washed once with PBS, and flagella were sheared for 1 min using a vortex, resuspended in 20 ml of PBS, kept on ice for 5 min, and spun down at 22,000 rpm using a Beckman ultracentrifuge with a T70i fixed-angle titanium rotor for 90 min. The pellets were resuspended in 200 μl of 1x Laemmli buffer in PBS. Samples were heated for 5 min at 60°C before loading onto an 8% SDS-PAGE gel.

### Abiotic Surface Attachment Assay

For the abiotic surface attachment assay, *A. brasilense* Sp7 strain and its *cheY* mutant derivatives carrying the pHRGFP plasmid (Ramos et al., 2002) were cultured overnight in TY medium supplemented with tetracycline for plasmid maintenance. Cells were washed with a chemotaxis buffer and resuspended in the chemotaxis buffer to a final OD_600_ = 0.4. Economy Plain Glass Microscope Slides Glass slides (Fisher Scientific™) were covered with 0.01% poly-lysine (Sigma™) and left to dry for 15 min. Ten microliters of cell cultures were placed on the slide and kept in a humidity chamber (square Petri dishes lined up with Kim Wipes wetted with sterile water) to prevent buffer evaporation. Cells remaining on the slide were washed with chemotaxis buffer 2 h after inoculation and imaged using a GFP filter, 4x objective mounted to a Nikon ECLIPSE 80i fluorescence microscope with a Nikon CoolSnap HQ2-cooled charge-coupled device (CCD) camera.

### Biofilm Formation

Biofilm assay was performed in modified MMAB medium modified to achieve a C:N ratio = 2 using fructose at 27.6 and 13.8 mM KNO3 as N source (Arruebarrena Di Palma et al., 2013). Two hundred microliters per well were transferred to sterile, clear flat-bottom polystyrene 96-well plates (Corning™) and incubated without agitation for 96 h at 28°C. Biofilm formation was determined using crystal violet staining (Arruebarrena Di Palma et al., 2013). Briefly, 200 μl of 0.5% crystal violet was added to each well, followed by incubation 30 min at room temperature, and then washed carefully three times with tap water. Crystal violet remaining attached to the wells was extracted with 200 μl of 33% v/v acetic acid. The OD_590_ of supernatants was determined using a microplate Absorbance Reader with Gen5 software (BioTek Instruments, Winooski, Vermont, United States). Data were normalized by total growth estimated by OD_600_ measured on the planktonic culture.

### Wheat Root Attachment Assay

*Triticum aestivum* cv. Jagger (wheat) seeds were utilized throughout this study. *T. aestivum* seeds were surface-sterilized 10 min with 90% ethanol and 20 min with a sterilization buffer containing 1% Triton X-100, 10% bleach and sterile water. After sterilization, seeds were planted into c (0.132 g/l CaCl_2_, 0.12 g/l MgSO_4_ 7H_2_O, 0.1 g/l KH_2_PO_4_, 0.075 g/l Na_2_HPO_4_

2H_2_O, 5 mg/l Fe-citrate, and 0.07 mg/l each of MnCl_2_ 4H_2_O, CuSO_4_ 5H_2_O, ZnCl_2_, H_3_BO_3_, and Na_2_MoO_4_ 2H_2_O, adjusted to pH 7.5 before autoclaving; Zamudio and Bastarrachea, 1994; de Oliveira Pinheiro et al., 2002; Greer-Phillips et al., 2004) and placed in the dark for 48 h to germinate. Next, seedlings were placed in 250 ml Mason jars containing 50 ml of semi-solid (0.5% w/vol Noble agar) Fahraeus medium and allowed to grow with 8 h day/16 h dark at 22°C in the plant growth chamber at 90,000 lux or 1,670 μmol m^−2^ s^−1^. All assays were performed on germinated and surface-sterilized seedlings that were 7–10 days old.

For the root attachment assay, *A. brasilense* strains were cultured in MMAB liquid overnight (28°C, 200 rpm). The cultures were normalized to an OD_600_ = 0.6 using sterile chemotaxis buffer and resuspended in 2 ml of Fahraeus medium in a 15 ml Falcon tube. Wheat seedling with cut-off leaves was placed inside the tube, and tubes were incubated either at room temperature for 2 h or with shaking on a Ferris wheel for 2 h. After incubation, roots were washed three times with sterile chemotaxis buffer, resuspended in 2 ml sterile chemotaxis buffer and sonicated for 10 s, using a cell dismembrator (Model 100; Fisher Scientific™, Waltham, MA, United States). CFU recovered from the inoculum or after detachment from roots were counted by plating serial dilutions on TY plates supplemented with ampicillin. The results were expressed as a root attachment index, calculated as CFU detached from roots/CFU in the initial culture normalized to the total fresh weight of roots in milligrams.

### Statistical Analysis

We used Student *t*-test using GraphPad Prism (version 8) software (GraphPad Software Inc., San Diego, CA, United States) to compare the wild type and mutant phenotypes (swimming/swarming behavior on the plates, band quantitation, western blotting flocculation, biofilm formation, and plant root attachment).

## Results

### *Azospirillum brasilense* CheY6 and CheY7 Response Regulators Have Different Contributions to Aerotaxis

**Figure 2:**
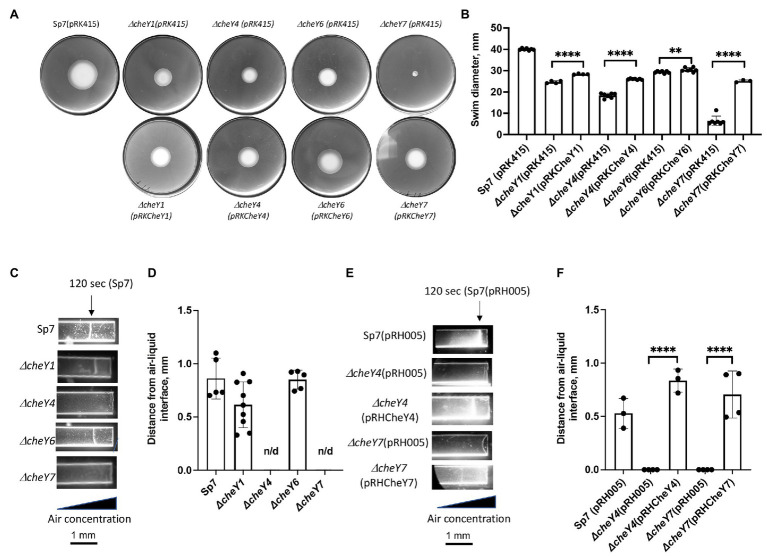
Detection and quantification of chemotaxis **(A,B)** and aerotaxis **(C-F)** responses in the *A. brasilense* and *Δ*cheY mutant derivatives. **(A)** Chemotaxis in the soft agar plate assay. **(B)** Quantification of chemotaxis in the soft-agar plate assay. **(C)** Aerotaxis in the spatial gradient capillary assay. The air gradient is established by diffusion into capillary tubes filled with a suspension of motile cells. The triangle represents decreasing air concentration as a result of diffusion within the cell suspension. The images were taken 120 s after placement of the cells into the capillary tubes. **(D)** Quantitation of the aerotaxis response represented as the distance from the edge of the air-liquid interface. **(E)** Aerotaxis of Sp7 (pRH005), *ΔcheY4* (pRH005), *ΔcheY7* (pRH005), and mutants complemented with CheY4-YFP, expressed as pRHCheY4 (for *ΔcheY4*) and CheY7-YFP, expressed as pRHCheY7 (for *ΔcheY7*) in the spatial gradient assay for aerotaxis. **(F)** Quantitation of the aerotaxis response in Sp7(pRH005), *ΔcheY4* (pRH005), *ΔcheY7* (pRH005), and mutants complemented with CheY4-YFP, expressed as pRHCheY4 (for *ΔcheY4*) and CheY7-YFP, expressed as pRHCheY7 (for *ΔcheY7*) in the spatial gradient assay represented as the distance from the edge of the air-liquid interface. ^**^p < 0.01 and ^****^p < 0.001.

Our previous work has shown that minor (*ΔcheY1* and *ΔcheY6* mutants) and severe (*ΔcheY4* and *ΔcheY7* mutants) defect in chemotaxis of the mutant strains could be functionally rescued by expressing parental genes from broad host range vectors (Bible et al., 2008, 2012; Mukherjee et al., 2016, 2019). Here, we confirm these previous observations by analyzing chemotaxis in swim plate assays (Figure 2A). The *ΔcheY1*, *ΔcheY4*, *ΔcheY6*, and *ΔcheY7* mutants had slight or major chemotaxis defects that were partially complemented by expressing *parental genes* expressed from broad host range plasmids (Figures 2A,B). Partial functional complementation from this type of plasmids was reported previously (Bible et al., 2008). We also performed the aerotaxis assay with the mutant strains. Aerotaxis is a particular form of chemotaxis in air gradients and is regulated by the same chemotaxis pathways. Aerotaxis depends on near-immediate responses to the air gradient (~1–2 min) instead of the several days for observing a response in chemotaxis spatial gradient assays. Aerotaxis thus provides a more direct evaluation of the ability of cells to navigate gradients. In the aerotaxis gradient assay, an open-ended capillary tube is filled with a suspension of motile *A. brasilense* cells. An air gradient is established through the diffusion of air from the atmosphere into the cell suspension (Zhulin et al., 1996; Alexandre et al., 2000). Under these conditions, motile and chemotaxis-competent *A. brasilense* cells form a tight band of motile cells at a location in the gradient that corresponds to maximal energy generation (Zhulin et al., 1996; Figures 2C–F). Chemotaxis defects of the different *cheY* mutant strains were also complemented by expressing parental genes from plasmids (Figures 2A,B,E,F).

### The Absence of *CheY7* Causes Defects in Polar Flagellin Molecular Weight but Not Swimming Motility

**Figure 3:**
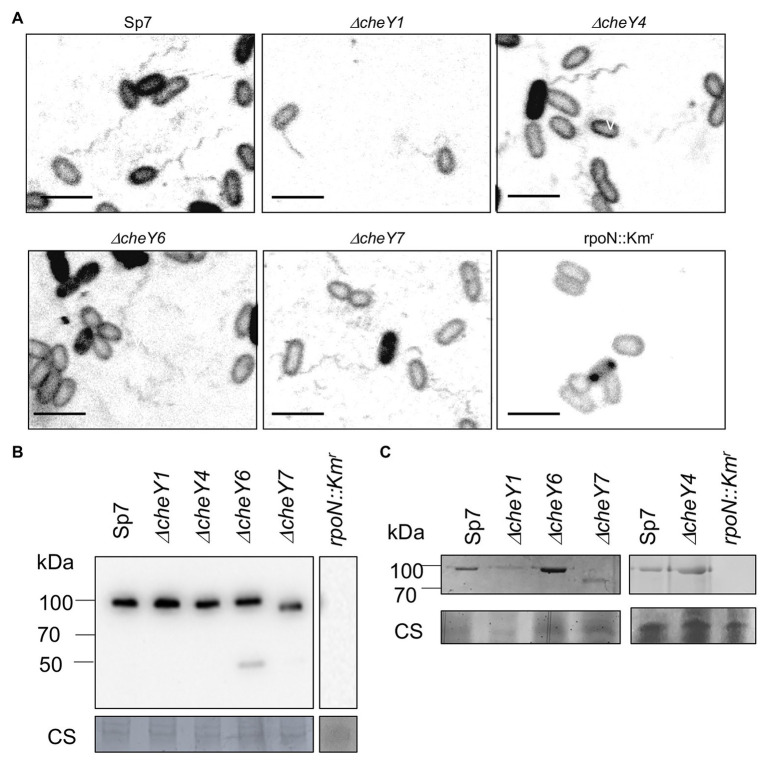
Visualization of the polar flagella in *A. brasilense* strains. **(A)** Representative images of Sp7, *ΔcheY1*, *ΔcheY4*, *ΔcheY6*, and *ΔcheY7* cells with polar flagella stained with Alexa fluor™ 488 NHS Ester. A *rpoN::Km^r^* mutant which is aflagellate and non-motile was used as a negative control. Images are presented as the negatives of the original images. Scale bar = 5 μm. **(B)** Western blot analysis with anti-polar flagellin (1:1,000) polyclonal antisera of whole-cell preparations of *A. brasilense* Sp7, *ΔcheY1*, *ΔcheY4*, *ΔcheY6*, *ΔcheY7*, and *rpoN::Km^r^* mutants grown in liquid media. Coomassie staining is shown below for an evaluation of loading control. **(C)** Glycoprotein staining of polar flagellin produced by wild type Sp7 and *ΔcheY1*, *ΔcheY4*, *ΔcheY6*, and *ΔcheY7* cells *ΔcheY1*, *ΔcheY4*, *ΔcheY6*, and *ΔcheY7* mutants. *rpoN::Km* strain was used as the negative control. The black lines indicate junctions separating lanes that were spliced from the original image in order to show samples run on the same gel in a single row.

All of the mutants lacking CheY1, CheY4, CheY6, or CheY7 are motile in liquid media and, as expected, possess polar flagella (Figure 3A). We used polyclonal antisera raised against the polar flagellin and Western blots to compare levels of production of the polar flagellin in the wild type Sp7 and its *cheY* mutant derivatives (Figure 3B). As a negative control, we used a *rpoN::Km^r^* mutant which lacks both polar and lateral flagella production (Milcamps et al., 1996; Figures 3A,B). As expected, the anti-polar flagellin antisera recognized a band at a predicted ~100 kDa in all but the *ΔcheY7* mutant. In this mutant, the band corresponding to the polar flagellin migrated with an apparent molecular weight of ~90 kDa. A second band at about 45–50 kDa was also observed in the *ΔcheY6* mutant. This molecular weight is much lower than the predicted molecular weight for the two polar flagellins encoded in the *A. brasilense* genome (AMK58_10890 and AMK58-18185), which are expected to be about 65 kDa. The nature of this cross-reacting band is not known. The polar flagellin is glycosylated in *A. brasilense* and a fully glycosylated polar flagellin has a molecular weight of about 100 kDa, while complete chemical deglycosylation of the polar flagellin yields a band at ~65–70 kDa (Moens et al., 1995b). Therefore, we hypothesized that changes in the molecular weight of the polar flagellin in the *ΔcheY7* strain could result from reduced glycosylation of the polar flagellin. Analysis of glycosylated proteins from the same samples as those used for the Western blot above, identified a single band for a glycosylated protein at the same molecular weight as the polar flagellin for all strains except for the non-flagellated *rpoN::Km^r^* strain (Figure 3C). Noticeably, a glycosylated band corresponding to the polar flagellin of the *ΔcheY7* mutant was still present at a lower molecular weight (Figure 3C). The reduced molecular weight of the polar flagellin of the *ΔcheY7* mutant could suggest reduced glycosylation levels in this species. A *ΔcheY7* mutant is still fully motile, so this different glycosylation level has no apparent effect on polar flagellum function. This observation is intriguing because it suggests a functional link between CheY7 and polar flagellin maturation.

### *Azospirillum brasilense* Chemotaxis CheY Homologs Contribute to Movement in Media of Varying Viscosity

**Figure 4:**
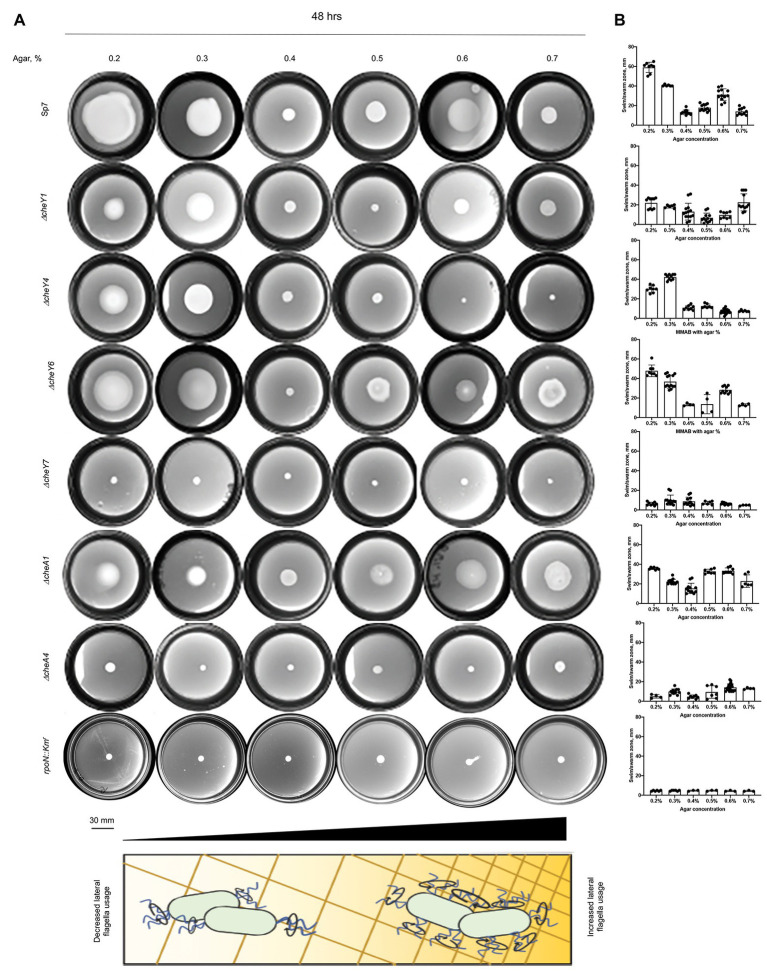
Navigation of *A. brasilense* and its chemotaxis mutant derivatives in soft agar media of varying viscosity. **(A)** Chemotaxis behavior of *A. brasilense* Sp7, *ΔcheY1*, *ΔcheY4*, *ΔcheY6*, *ΔcheY7*, *ΔcheA1*, *ΔcheA4*, and *rpoN::Km^r^* strains in the semi-solid medium supplemented with 0.2–0.7% agar. The triangle at the bottom and the scheme represents increasing concentrations of agar. **(B)** Quantitation of the swimming/swarming ring diameters. Arrows indicate a significantly higher swimming/swarming diameter of the colonies in the mutant strains than Sp7. Student *t*-test was used to determine significance between Sp7 and mutants. ^*^p < 0.05, ^**^p < 0.01, ^***^p < 0.005, and ^****^p < 0.001. All studies were done in three biological replicates.

CheY homologs have different effects on the free-swimming motility pattern of *A. brasilense*: CheY1 affects the transient increase in swimming speed and has a minor impact on the probability of swimming reversals. In contrast, CheY4 and CheY6 have severe defects in swimming reversals, and CheY7 cannot reverse the swimming direction (Mukherjee et al., 2019). These different effects are hypothesized to provide *A. brasilense* with swimming navigation strategies optimized for the heterogeneous physicochemical conditions found in the soil. Here, we probe the chemotaxis response regulators’ role in controlling the swimming bias in modulating navigation in porous media by comparing the movement of cells under conditions of increasing agar concentrations (Figures 4A,B). We also included *ΔcheA1* and *ΔcheA4* mutants that are impaired or null for chemotaxis, respectively (Figures 4A,B; Bible et al., 2008; Mukherjee et al., 2016), and the *rpoN::Km^r^* mutant strain that is immotile and lacks flagella (Milcamps et al., 1996). At low agar concentrations (0.2–0.3%), *A. brasilense* cells are swimming through the medium using their polar flagellum, given the abundance of water under these conditions and previous observation by others (Hall and Krieg, 1983; Moens et al., 1995b; Figure 4). At higher agar concentrations (0.6–0.7%), wild type cells are preferentially swarming using lateral flagella, given that they are observed to move on top of the medium and that these agar concentrations were previously described as optimum for swarming for *A. brasilense* (Hall and Krieg, 1983; Moens et al., 1995b). Navigation of the wild type cells within or on top of the medium at intermediate agar concentrations (0.4%) was minimal, suggesting that both swimming and swarming are limited under these conditions (Figures 4A,B).

Compared to the wild type, the *rpoN::Km^r^* mutant did not expand beyond the inoculation point, regardless of agar concentrations, a behavior mimicked by the chemotaxis null mutants *ΔcheA4* and *ΔcheY7*. The *ΔcheY4* mutant had reduced expansion at low agar concentrations (0.2–0.5%) and did not expand beyond the site of inoculation at higher agar concentrations (Figures 4A,B). The *ΔcheY4* mutant is non-chemotactic because it seldom reverses swimming direction and this strain also has an elevated frequency of swimming pauses (Mukherjee et al., 2019). We surmise that the limited expansion of the *ΔcheY4* cells in media with low agar concentrations is related to these cells’ ability to pause with a high frequency during swimming, which would allow them to escape entrapment into the agar polymers. This swimming bias would not be sufficient to promote movement at higher agar concentrations (0.6–0.7%). The mutants that are impaired but not null for chemotaxis (*ΔcheA1*, *ΔcheY1*, and *ΔcheY6*) displayed either no or only minor defects in navigating within or on top of media of different viscosity. The *ΔcheA1* mutant had a marginally increased expansion through media at the highest agar concentration tested (Figures 4A,B). Together, these data indicate that chemotaxis is essential for the ability of cells to navigate media of varying viscosity, including across surfaces by swarming. Chemical gradients are produced by metabolism as cells move through the agar media and grow indicating that the existence of a gradient is sufficient to trigger an expansion in viscous media by either swimming or swarming. The data presented here confirm the hypothesis that *ΔcheY1* and *ΔcheY6* mutants are functionally linked to *ΔcheA1*, while *ΔcheY4* and *ΔcheY7* cells are related to *ΔcheA4* mediated signaling.

**Figure 5:**
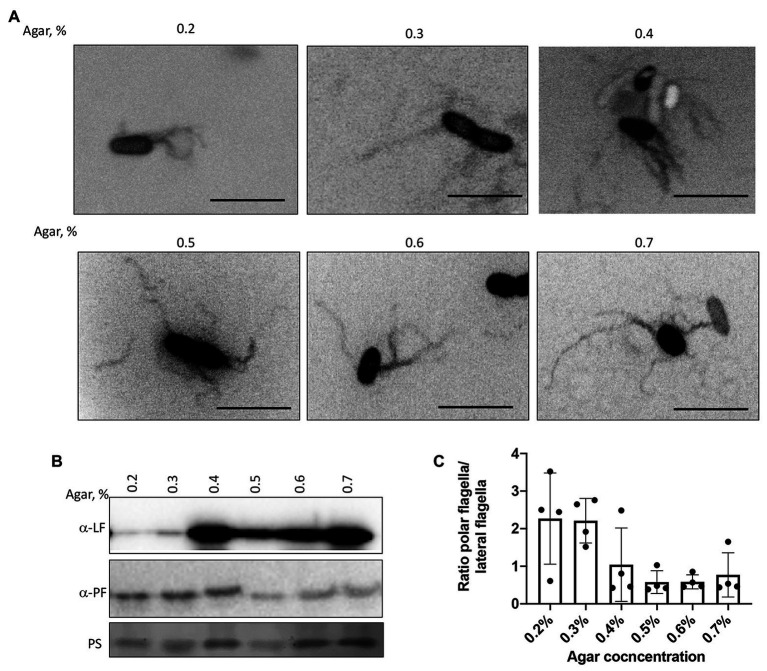
Visualization of the polar and lateral flagella in *A. brasilense* strains. **(A)** Representative images of Sp7 cells collected from the media with 0.2–0.7% agar and stained with Alexa fluor™ 488 NHS Ester. Arrows indicate localization of the polar flagella. Images are presented as the negatives of the original images. Scale bar = 5 μm. **(B)** Western blot analysis with anti-polar (1:1,000) and anti-lateral flagellin (1:1,000) polyclonal antisera of whole-cell preparations of *A. brasilense* Sp7 collected from the media with 0.2–0.7% agar. Ponceau staining (PS) is shown below for evaluation of loading control. **(C)** Ratio between polar and lateral flagellin abundance in wild type cells grown in media solidified with 0.2–0.7% (w/vol) agar. Quantitation was performed based on the Western blotting analysis *p*-values.

We next confirmed the type of motility employed by the cells under these conditions by visualizing the wild type strain’s flagellation when inoculated in semi-soft media at different agar concentrations (Figure 5A). At 48 h post-inoculation, both polar and lateral flagella were visible regardless of agar concentration (Figure 5A). Lateral flagella are not produced constitutively in contrast to the polar flagellum in *A. brasilense*. However, we observed some wild type cells with lateral flagella, even at low viscosity [0.2% (v/v) of agar]. This suggests that conditions in the soft agar plates are heterogeneous and do not perfectly replicate a single motility type. To confirm these observations, we used polyclonal antisera raised against the polar and lateral flagellins and Western blots (Figures 5B,C). As expected, the anti-polar flagellin antisera recognized a single band at ~100 kDa in cells grown at various viscosity. The most significant induction of the lateral flagellin production, relative to the polar flagellin production, was observed starting at 0.4% agar in the medium and remained elevated at higher viscosity conditions (Figures 5B,C). Concurrent to these changes, we also observed that cell size distribution varied depending on agar concentrations. The wild type cells were the shortest when incubated in 0.2% agar plates. They tended to increase in size when cells were incubated in media with 0.3–0.6% agar and became shorter at 0.7% agar. However, cells from 0.7% agar were longer than cells at 0.2% agar (Supplementary Figure 1). The longer cell sizes roughly match the induction of lateral flagellin production under these conditions, suggesting that cell elongation is concomitant with swarming. These findings are consistent with similar differentiation observed in other species (Kim et al., 2003; Skerker and Laub, 2004; Little et al., 2019).

**Figure 6:**
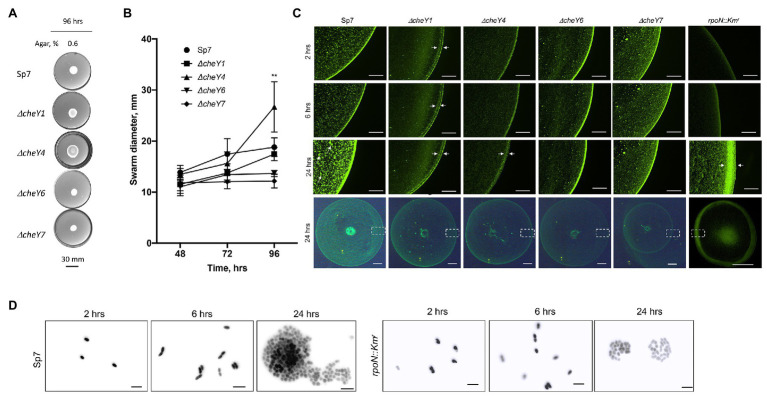
Time course of swarming of *A. brasilense* and its chemotaxis response regulator mutants. The plates were solidified with 0.6% (w/vol) agar and incubated for 96 h post-inoculation at 28°C. **(A)** Representative images of swarming for the strains tested. **(B)** Quantitation of the ring diameters of Sp7 and mutant strains after 24–96 h of incubation on the swarming medium. Student *t*-test was used to determine significance between Sp7 and mutant strains (^**^p < 0.01). All experiments were done in triplicate. **(C)** The appearance of the border of the swarming colony of Sp7, *ΔcheY1*, *ΔcheY4*, *ΔcheY6*, *ΔcheY7*, and *rpoN::Km* strains harboring pHRGFP plasmid. The edge of the colony was photographed at 2, 6, and 24 h. The scheme at the bottom represents the area photographed. Scale bar: colony border images is 0.45 mm, whole colony is 1 mm. Exposure time for the colony border imaging is 9 s, for the whole colony 6.6 s. Arrows indicate inner and outer swarming rings in colonies of the *ΔcheY1* and *ΔcheY4* strains and cell clusters in the Sp7 swarming colony. **(D)** Visualization of the cell cluster formation by Sp7 (left panel) and *rpoN::Km*^r^ cells in time. Scale bar = 5 μm.

To determine whether the defects were due to lack of swarming vs. delayed swarming, we also compared *ΔcheY1*, *ΔcheY4*, *ΔcheY6*, and *ΔcheY7* mutants with the wild type Sp7 strain for swarming over time. We performed the experiments using MMAB medium solidified with 0.6% noble agar and supplemented with Tween-20, corresponding to conditions that permit robust swarming (Figures 6A,B). Compared to the wild type strain, all mutants had reduced swarming over the 96 h time-course experiment except for the *ΔcheY4* strain, which increased swarming at 96 h post-inoculation, following a reduced swarming at other time points (Figures 6A,B). The *ΔcheY4* strain also appeared to expand into a larger swarm colony than the wild type strain under these conditions. This result indicates that the *ΔcheY4* strain, but not the other strains, is delayed in inducing swarming. Collectively, the data suggest that CheY7 is essential for swarming and that CheY4 is necessary for timely swarming.

The surface of swarming colonies also differ between the strains: the swarming colonies of the wild type, the *ΔcheY6*, and *ΔcheY7* mutants had a homogenous surface appearance while the swarming colonies of the *ΔcheY1* and *ΔcheY4* displayed a thick front edge and a thinner, less dense inner region, suggesting that the reduced swarming of the strains could be caused by different group behaviors within the swarming colony. To further characterize cells’ behavior in swarming colonies, we next labeled cells with a constitutively expressed GFP from a plasmid (Ramos et al., 2002) and inoculated these on top of a swarming agar pad. We used fluorescent microscopy to observe cellular behavior at the initiation of swarming (first 24 h). Under these conditions, the wild type cells appeared to organize into a dense edge at the expanding front of the swarming colony, while cells formed clusters that grew in density over time behind this front (Figure 6C). A thick edge was seen in the *rpoN::Km^r^* mutant that is non-motile and non-flagellated, suggesting that it corresponds to non-motile cells. Still, the density of cells under these conditions did not change behind this front in the mutant, suggesting that motility is required for this organization (Figure 6C). A major difference was evident between the wild type swarming colony and the *cheY* mutants: no increasing clustering of cells behind the expanding front was observed in any of the mutants (Figure 6C). This observation indicates that the organization of cells as high-density clusters is required for the ability of a swarming colony to expand on a surface. Given the differences in the *cheY* and *rpoN::Km^r^* mutant strains, the lack of cellular clustering suggests that swimming motility, a polar flagellum able to reverse, change swimming speed, and pause is essential for this behavior. A second difference was that the *ΔcheY1* and *ΔcheY4* swarming colony’s edge was thicker and diffuse compared to that of the wild type or the other mutants. In contrast, the *ΔcheY6* and *ΔcheY7* swarm colonies had a uniform and bright expanding edge that did not thicken over time compared to that of the wild type (Figure 6C). When compared to the behavior of the *rpoN::Km*^r^ mutant under these conditions, the *ΔcheY6* and *ΔcheY7* mutant strains may differ in the timing or proportion of cells losing motility that produces the edge of the swarm under these conditions compared to the *ΔcheY1* and *ΔcheY4* or wild type swarming colonies (Figure 5C). In all *cheY* and the *rpoN::Km*^r^ mutants, cell density was noticeably reduced behind this expanding edge. Consistent with the results above, the size of the swarming colonies at 24 h was similar in all the mutants, except for the *ΔcheY7* and the *rpoN::Km*^r^ mutants, which produced significantly smaller swarms at 24 h post-inoculation under these conditions (Figure 6C). These results suggest that swarming requires that cells be able to form high-density clusters behind a sharp and dense expanding edge that may be composed of non-motile cells. When observed under high magnification (Figure 6D), cells behind the expanding edge appeared to organized as growing clusters that adopt a three-dimensional organization in the wild type but not in the immotile *rpoN::Km*^r^ mutant, suggesting a role of motility for these clusters (Figure 6D). Given that some of the mutants are still able of chemotaxis (CheY1, CheY6) while others are chemotaxis null (CheY4 and CheY6), these results indicate that chemotaxis *per se* is not required for the formation of these clusters but that functional CheY response regulators are essential for this behavior. Together, our data indicate that chemotaxis underlies the expansion of a swarming colony across media of high viscosity and that motility and functional CheYs, but not chemotaxis *per se*, is required for initial cell-cell interactions and clustering within a swarming colony.

### Lateral Flagella Are Produced in All but the *ΔcheY7* Mutant

**Figure 7:**
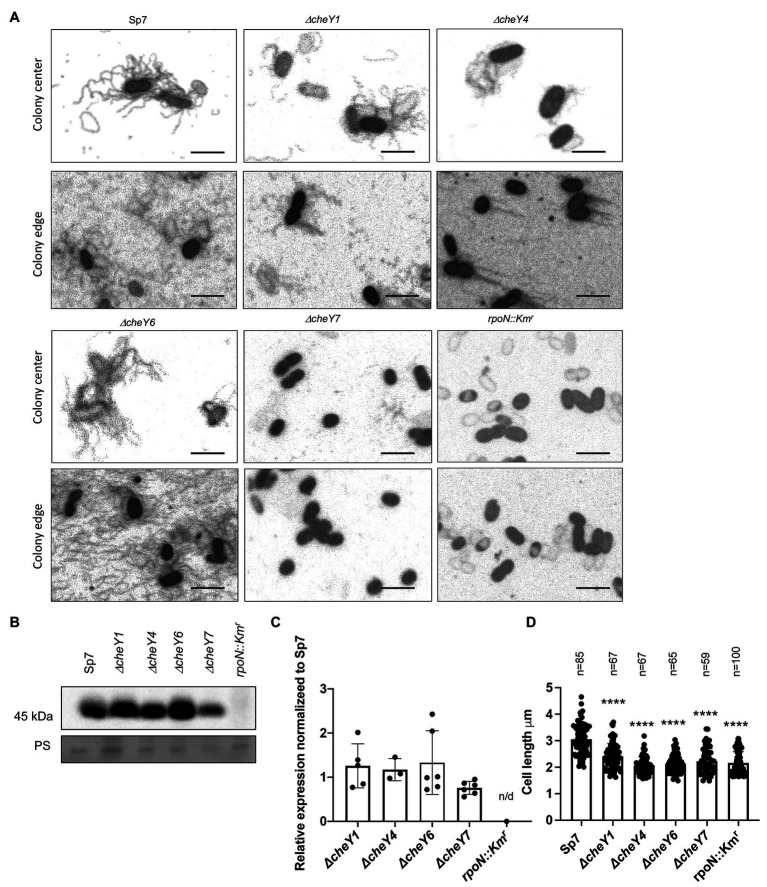
Detection of lateral flagella produced in swarming colonies of *A. brasilense* Sp7 and mutant response regulator strains. **(A)** Representative images of separate cells within and at the edge of a swarming colony. Arrows indicate lateral flagella. **(B)** Western blot analysis with anti-lateral flagellin (1:1,000) polyclonal antisera of whole-cell preparations of *A. brasilense* Sp7, *ΔcheY1*, *ΔcheY4*, *ΔcheY6, ΔcheY7,* and *rpoN::Km*^r^ mutant strains. Coomassie staining is shown below for the loading control. **(C)** Quantitation of relative lateral flagellin levels normalized to levels in the wild type Sp7 strain. Student *t*-test was used to determine the significance between Sp7 and mutant strains. **(D)** Quantitation of the cell lengths of Sp7, *ΔcheY1*, *ΔcheY4*, *ΔcheY6*, and *ΔcheY7* mutant strains grown under swarming conditions. Student *t*-test was used to determine significance between Sp7 and mutant strains (^****^p < 0.005).

Swarming depends on the production of lateral flagella, prompting us to analyze lateral flagella and flagellin production in the chemotaxis mutants (Figures 7A–C). Flagella staining revealed that lateral flagella were abundant at 24 h post-inoculation in the *ΔcheY1*, *ΔcheY4*, and *ΔcheY6* mutants, less abundant in the *ΔcheY7* mutant and, as expected, absent in the *rpoN::Km*^r^ mutant (Figures 7A–C). This suggests that the reduced swarming of the *cheY* mutant strains is not due to the inability to induce lateral flagella production. Next, we used polyclonal antisera raised against the lateral flagellin and Western blots to compare lateral flagellin production in the wild type and the chemotaxis mutant strains with the immotile *rpoN::Km*^r^ strain as a negative control (Figures 7B,C). As expected, the anti-lateral flagellin antisera recognized a single band, at ~45 kDa, in all strains, except the *rpoN::Km*^r^ mutant. Relative to the wild type Sp7 strain, the *ΔcheY7* mutant, but not the other strains, had a significantly lower abundance of lateral flagellin, consistent with our observations from flagella staining (Figure 7A). Thus, the inability of strain *ΔcheY7* to swarm is likely related to its reduced lateral flagellin production. The other mutants’ reduced swarming is not related to defects in the production of lateral flagella or lateral flagellin.

Compared to the wild type, swarming cells of the *ΔcheY1*, *ΔcheY4*, *ΔcheY6*, and *ΔcheY7* and the *rpoN::Km*^r^ mutant strains were shorter in length compared to the wild type, consistent with their defective swarming (Figure 7D). Together, these data suggest that productive swarming requires lateral flagellin production and cell elongation in *A. brasilense* and that cells with defective swarming also display defective cell elongation. These observations are consistent with observations by others that productive swarming requires cell elongation and increased lateral flagella production (Little et al., 2019). Together, the data indicate that the chemotaxis mutants studied here can induce lateral flagella and differentiate into swarmer cells, albeit at different levels. The reduced (or lack of) swarming of these mutants is not due to an inability to produce cellular structures required for swarming.

### Functional CheY Homologs, but Not Chemotaxis, Contribute to Attachment to Abiotic and Root Surfaces

**Figure 8:**
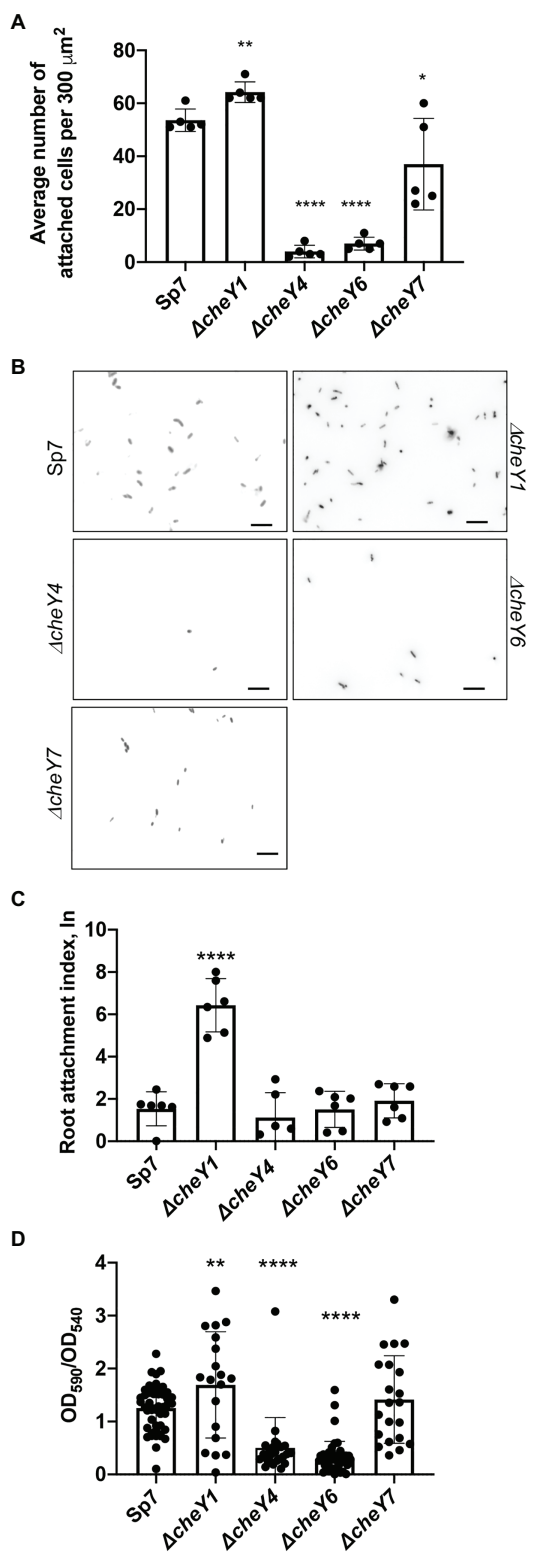
*Azospirillum brasilense* wild type and *cheY* mutant strains attachment to abiotic and biotic surfaces and biofilm formation. **(A)** Quantitation of cell attachment of the Sp7, *ΔcheY1*, *ΔcheY4*, *ΔcheY6*, and *ΔcheY7* strains labeled carrying pHRGFP, which constitutively expresses green fluorescent protein (GFP), to glass slides coated with poly-lysine (^*^p < 0.05, ^**^p < 0.01, and ^****^p < 0.001). **(B)** Examples of images of the Sp7, *ΔcheY1*, *ΔcheY4*, *ΔcheY6*, and *ΔcheY7* cells attached to poly-lysine coated glass slides. Scale bar is 10 μm. **(C)** Quantitation of cell attachment of the Sp7, *ΔcheY1*, *ΔcheY4*, *ΔcheY6*, and *ΔcheY7* strains carrying pHRGFP, which constitutively expresses GFP, to sterile wheat roots. Student *t*-test was used to determine significance between Sp7 and mutant strains (^****^p < 0.005). **(D)** Quantitative biofilm formation by Sp7, *ΔcheY1*, *ΔcheY4*, *ΔcheY6,* and *ΔcheY7* cells formed in 96 h. Student *t*-test was used to determine significance between Sp7 and mutant strains (^**^p < 0.01 and ^****^p < 0.005).

Given the previously reported role of the polar flagellum in an initial attachment to a surface (about 2 h; Michiels et al., 1991), we next compared CheY homologs for contribution to attachment to abiotic surfaces. We used a wild type Sp7, *ΔcheY1*, *ΔcheY4*, *ΔcheY6*, and *ΔcheY7* strains harboring pHRGFP plasmid (Ramos et al., 2002) to monitor attachment to poly-lysine coated glass slides over a 2-h incubation period. Relative to the wild type, the *ΔcheY1* cells attached better while the Δ*cheY4*, Δ*cheY6*, and to a lesser extent, *ΔcheY7* cells, attached significantly less to abiotic surfaces (Figures 8A,B).

A similar pattern of attachment to sterile wheat roots was observed (Figure 8C), with the *ΔcheY*1 attaching better to wheat roots within a 2-h incubation compared to the wild type or the other mutant strains.

We also monitored formation of biofilms *in vitro* and detected major differences at 96 h post-inoculation. The *ΔcheY1* strain formed more biofilms relative to the wild type strain, and the *ΔcheY4* and *ΔcheY6* strains formed less biofilm. The *ΔcheY7* mutant did not display any significant defect in biofilm formation relative to the wild type (Figure 8D). The data obtained here in abiotic surface and root attachment and biofilm formation are in good agreement and indicate that CheY homologs are required for attachment and biofilm formation. However, the differences between the strains are unrelated to their steady-state swimming bias or their chemotaxis abilities. These results suggest that functional chemotaxis, which is absent in strains lacking CheY4 or CheY7 and present in strains lacking CheY1 and CheY6, is not directly implicated in these behaviors. The results also imply that the control of the polar flagellum rotation by CheY homologs triggers distinct attachment behaviors.

## Discussion

Here, we show that the four CheY homologs that regulate the polar flagellum rotational bias in *A. brasilense* and differentially alter chemotaxis and aerotaxis responses have distinct effects on swarming and attachment to surfaces. Specifically, we show that chemotaxis signaling, mediated by the CheY homologs’ activity studied here, is required for the ability of colonies to expand within or atop media of varying viscosity, likely in response to gradients generated by cell metabolism during this movement. This is similar to findings reported for the role of chemotaxis in mediating expansion of swarming colonies in gradients in other dually flagellated bacteria such as *Vibrio parahaemolyticus* (Sar et al., 1990), *Vibrio alginolyticus* (Kojima et al., 2007), and *Rhodospirillum centenum* (Jiang et al., 1997). However, this role for chemotaxis in promoting the expansion of swarming colonies is not shared by other species, which increased production of a single type of flagella during swarming, such as *E. coli* (Burkart et al., 1998) or *Bacillus subtilis* (Kearns and Losick, 2003).

Functional CheYs appear required for the organization of cells in clusters that formed behind the moving edge of a swarming colony in *A. brasilense*. The cell clusters observed in the wild type are reduced or absent in the *cheY* mutant strains. Our data suggest that the formation of these clusters contributes to productive swarming in *A. brasilense* since swarming is diminished or absent in the chemotaxis *cheY* mutants, despite their ability to induce lateral flagella production. However, we recognize that our experimental design was limited and that additional mutants and higher-resolution imaging are needed to conclude on their role during swarming expansion. We note that these clusters could resemble groups of cells, named rafts or packs that have been seen in other bacterial species (Copeland and Weibel, 2009; Partridge and Harshey, 2013). In *E. coli* and *B. subtilis*, these rafts move together, form and reform continuously with collisions leading to realignment of cells along their long axis and the observation of swirling motions (Turner et al., 2010). The role of chemotaxis in mediating cell-to-cell interactions has been previously demonstrated in several motile bacteria, including *A. brasilense* (Bible et al., 2008; Alexandre, 2015), but the exact mechanism(s) are not known. In *E. coli* (Burkart et al., 1998) and other species that use a single type of flagellum for swimming and swarming, such as *B. subtilis* (Kearns and Losick, 2003) and *P. aeruginosa* (Overhage et al., 2008), a basal level of changes in swimming direction (tumble) triggered by chemotaxis signaling is required for proficient swarming, though the tumbling rate is significantly lower than that observed for free-swimming cells (Partridge et al., 2019). This behavior is thought to promote side-by-side cell alignments and coordinated movement of groups of cells within packs as the swarming colony advances (Partridge et al., 2019, 2020). The lateral flagella that power swarming in *A. brasilense* are structurally different from the polar flagellum, and these differences extend to flagellar motors composed of distinct proteins. We have no evidence that the lateral flagella reverse swimming direction during swarming in *A. brasilense* or that the CheY homologs studied here interact with lateral flagella motors. We only have experimental evidence these CheY homologs control the polar flagellum rotational bias (Mukherjee et al., 2019). The polar flagellum is constitutively produced and persists in swarming cells in *A. brasilense* (Hall and Krieg, 1983; Borisov et al., 2009), but its role is unclear. We have not measured motility and analyzed the motility bias directly from swarming cells. Therefore, how the CheY homologs exert their effects on swarming through control of the polar flagellum rotational bias remains to be elucidated.

The advancing edge of the *A. brasilense* swarming colony suggested it organized as a densely front of cells, similar to the swarm monolayers described in other bacterial species (Partridge and Harshey, 2013). The swarm colony’s moving edge appears to include non-motile cells, similar to observations made in other bacterial species (Copeland and Weibel, 2009). The CheY homologs had different effects on this organization: strains lacking CheY6 and CheY7 formed a sharp front of densely packed cells, while strains lacking CheY1 and CheY4 formed a less defined front. These strains have different swimming biases and chemotaxis abilities. These differences could suggest that the role of CheY in this organization is independent from their role during chemotaxis. A similar observation was previously described in *E. coli* (Burkart et al., 1998). Together, the data suggest that CheY homologs are somehow required for motility loss and the formation of an advancing edge of a swarming colony in *A. brasilense*.

Our data also indicate that both CheY4 and CheY7 play a major role in induction of lateral flagella production and initiation of swarming, with CheY7 being essential for this function and CheY4 being required for timely induction of swarming upon surface contact. This is in contrast to the chemotaxis-independent induction of swarmer cell differentiation in *R. centenum* (Jiang et al., 1997) and *V. parahaemolyticus* (Sar et al., 1990), which also possess two types of flagellar systems and somewhat similar to the role of chemotaxis in inducing swarmer cell differentiation in *E. coli* and other species using a single flagellar system for swimming and swarming. *ΔcheY4* and *ΔcheY7* cells are both non-chemotactic suggesting that chemotaxis is required for induction of lateral flagella production and swarming motility. This effect is unlikely to be directly related to the rotational bias of the polar flagellum since CheY6 and CheY4 provoke similar rates of swimming reversals of the polar flagellum but a strain lacking CheY6 shows a greatest defect in swarming differentiation (as observed by changes in cell lengths) compared to a strain lacking CheY4, which swarms at high rates after a prolonged delay. In *A. brasilense*, production of the lateral flagellin (Laf1) which is the major component of the lateral flagella and required for swarming is induced when rotation of the polar flagellum is hindered (Moens et al., 1996). An extracytoplasmic factor (ECF) sigma homolog was recently shown to be at the top of a regulatory cascade that negatively regulates lateral flagellin biogenesis in *A. brasilense* (Dubey et al., 2020). These findings, together, suggest that some form of envelope stress may be a triggering signal for induction of lateral flagella in *A. brasilense*. If this is the case, then our findings suggest that CheY7 and CheY4 play essential roles in this signaling event.

Similar to their role in swarming, our results indicate that the *A. brasilense* CheY homologs, but not chemotaxis, mediate short-term attachment to abiotic surfaces and wheat roots, with these effects leading to similar changes in initial biofilm formation. These effects did not correlate with chemotaxis ability or polar flagellum motor bias. We note that all chemotaxis mutants have lower swimming speed compared to the wild type in the absence of a gradient but they can transiently increase swimming speed in response to attractants, with the exception of a strain lacking CheY1 which is locked at low speeds (Bible et al., 2012; Mukherjee et al., 2016, 2019). A lower swimming speed for this strain would increase its residence time in proximity to surfaces, which could promote enhanced adhesion for this mutant relative to the other strains. Several lines of experimental evidence indicate that chemotaxis signaling regulates non-chemotaxis functions in *A. brasilense*, though the mechanism(s) are not known (Bible et al., 2008; Gullett et al., 2017; Ganusova et al., 2021). It is thus also possible that the *cheY* mutants have different cell surface properties which would modulate their ability to adhere to surfaces.

Collectively, the data obtained here suggest that multiple CheY homologs not only fine tune the rotational bias of flagellar motors and chemotaxis but also promote behaviors that depend on motility such as swarming and attachment to surfaces.

## Data Availability Statement

The original contributions presented in the study are included in the article/Supplementary Material, further inquiries can be directed to the corresponding author.

## Author Contributions

EG designed and conducted the experiments, analyzed the data, and wrote the manuscript. LV and TM designed and conducted the experiments, and analyzed the data. GA designed the experiments, analyzed the data, and wrote the manuscript. All authors contributed to the article and approved the submitted version.

### Conflict of Interest

The authors declare that the research was conducted in the absence of any commercial or financial relationships that could be considered as a potential conflict of interest.

## Supplementary Material

Supplementary Figure 1 Distribution of the cell sizes of *Azospirillum brasilense* Sp7 grown in the media solidified with 0.2–0.7% (w/vol) agar.
