# Supplementary figures and images for: Multiple CheY Proteins Control Surface-Associated Lifestyles of Azospirillum brasilense
Source: Front Microbiol. 2021 Apr 22;12:664826. doi: 10.3389/fmicb.2021.664826 (PMC8100600; doi:10.3389/fmicb.2021.664826)

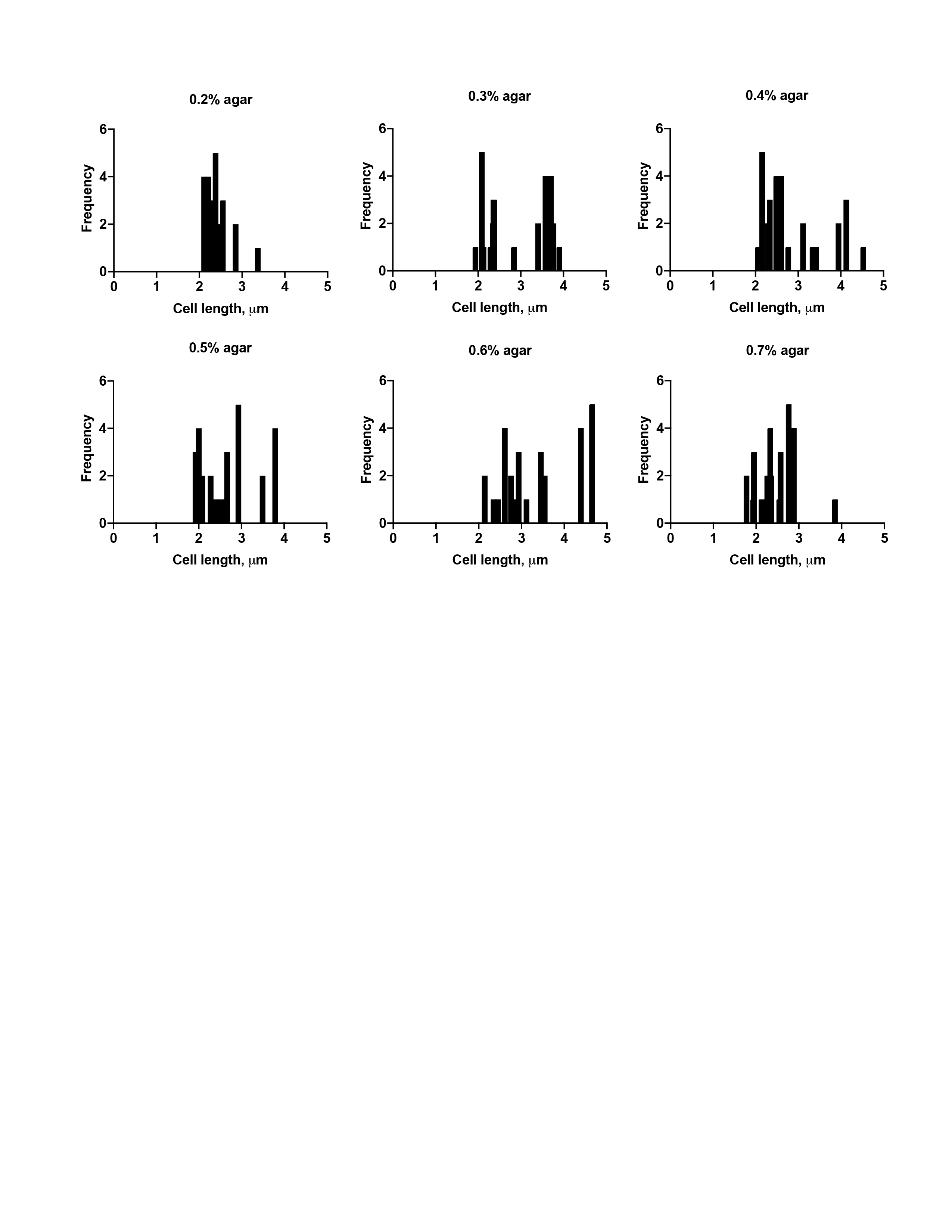

Supplement: Supplementary Figure 1 — Distribution of the cell sizes of Azospirillum brasilense Sp7 grown in the media solidified with 0.2–0.7% (w/vol) agar. [file Image_1.TIF]
